# The Rehabilitation Landscape in a Low-to-Middle-Income Country: Stakeholder Perspectives and Policy Implications—A Qualitative Study

**DOI:** 10.1177/00469580241271973

**Published:** 2024-10-07

**Authors:** Rentia Amelia Maart, Gubela Mji, Linzette Deidré Morris, Dawn Verna Ernstzen

**Affiliations:** 1 Division of Physiotherapy, Department of Health and Rehabilitation Sciences, Faculty of Medicine and Health Sciences, Stellenbosch University, Tygerberg, South Africa; 2 Centre for Rehabilitation Studies, Faculty of Medicine and Health Sciences, Stellenbosch University, Tygerberg, South Africa; 3 Department of Rehabilitation Sciences, College of Health Sciences, Health Sector, Qatar University, Doha, Qatar

**Keywords:** rehabilitation, stakeholders, South Africa, health care sector, policy, landscape, low-to-middle-income countries

## Abstract

The need for rehabilitation is increasing on a global level due to a rise in non-communicable diseases, aging and medical advances, and in South Africa (SA), due to the quadruple burden of disease. More information is required regarding rehabilitation scope and practices in SA to optimize the provision of rehabilitation interventions in the context of the transforming health care sector in SA, a low-to-middle-income country (LMIC). The purpose of this study is to explore the perspectives of South African rehabilitation stakeholders on the landscape of rehabilitation in SA. A descriptive qualitative study, with an interpretive approach, was used to explore stakeholder perspectives on rehabilitation practices in the public health care sector of SA. Semi-structured interviews were conducted with 12 rehabilitation stakeholders. Data were analyzed using a combination of deductive and inductive processes to generate themes and categories. We identified 5 main themes, with subsequent categories and sub-categories. The themes include a composite definition of rehabilitation, core elements of rehabilitation provision, challenges affecting rehabilitation practices, the importance of policy implementation, and the progress of rehabilitation in SA. Despite a common understanding of rehabilitation practices in SA amongst stakeholders, many persistent challenges hamper the delivery of effective rehabilitation services. We recommend that further research explore the rehabilitation needs of end-users, together with collaborative research for priority setting on the translation of policy to practice ensuring equitable and quality rehabilitation service delivery.

**1) What do we already know about this topic?**
Equitable, quality rehabilitation service delivery in low- and middle-income countries, such as South Africa, is often challenged by many barriers due to limited resources and the lack of efficient policy implementation.
**2) How does your research contribute to the field?**
The findings of this study confirm that South African rehabilitation stakeholders have a common understanding of and a shared vision for rehabilitation in South Africa which is important in driving the priorities for rehabilitation.
**3) What are your research’s implications toward theory, practice, or policy?**
The study reiterates that the common goal of providing quality and equitable rehabilitation in a resource-restrained South Africa can only be achieved through a collaborative and multisectoral approach.

## Introduction

The purpose of rehabilitation is the enablement of a person’s participation in meaningful life roles in different spheres of education, work, recreation, and family life. Any person with a health condition, who experiences limitations in their functioning may benefit from rehabilitation. Importantly, rehabilitation is essential in universal health coverage (UHC),^1^ where every person (and their communities) can access quality and equitable health care, including *preventive, promotive, curative, rehabilitative and palliative care*, without experiencing any financial hardship across the life course.^2^

Despite advances in rehabilitation practice and interventions, the global need for rehabilitation has increased by 63% since 1990^3^ and is going largely unmet.^1^ The reason why “*2.41 billion people could benefit from rehabilitation services*” has been attributed to population growth and aging,^3^ and an increase in the prevalence of non-communicable diseases (NCDs).^4,5^ Health care system factors such as a poorly integrated primary health care (PHC) approach, rehabilitation workforce challenges, limited health care financing as well as a lack of leadership and governance also play a role in the unmet need.^6^ In recognition of the substantial need for rehabilitation across the world, but especially in low- and middle-income countries (LMICs), the WHO launched “Rehabilitation 2030” which is a global call for action toward strengthening rehabilitation in health care systems, to achieve the goal of “health care for all.”^1,4^ In addition, the United Nations Convention on the Rights of People with Disabilities (UNCRPD) emphasizes the need to strengthen, organize and create comprehensive rehabilitation.^7^ Rehabilitation challenges are not unique to the South African context. Still, they are global concerns, which implies that global action from stakeholders within and across sectors is necessary to achieve Sustainable Development Goal (SDG) 3 of health care for all.

Acknowledging the diverse health challenges in the country^6^ and in pursuit of UHC, the South African government is in the process of major health systems restructuring by implementing a National Health Insurance (NHI). The NHI’s goal is to provide quality and equitable health care and ensure health care for all. This is a much-needed shift as the health care expenditure in South Africa, an upper-middle-income country, is unequal and fragmented.^8,9^ Currently, the South African health care system consists of the public health care sector (serving >80% of the population) as well as the private health care sector which provides health care to the minority of the population who can afford to belong to medical schemes or pay out-of-pocket health care costs.^10,11^ Each province in South Africa has its own legislature, premier and executive council,^12^ whereas the National Department of Health (NDoH) governs health care in South Africa. The role of the NDoH is policy formulation, monitoring and evaluation and provision of support and coordination to the Provincial DOHs.^13^ Each Provincial DOH is mandated to organize and deliver health care services to the province.^14^

In South Africa, health care, including rehabilitation, is provided at all levels of care which entails PHC services and health care provided at district, regional, tertiary and central hospitals, each with a specific purpose.^15-17^ The National Rehabilitation Policy (NRP)^15^ as well as the Framework and Strategy for Disability and Rehabilitation Services in South Africa (FSDR)^16^ are the guiding national legislative documents for rehabilitation services in South Africa, although limited guidance is provided in terms of rehabilitation service delivery within the NHI policy.^9^ However, despite the guidance from different national and international health care policies, access to and participation in rehabilitation services, remain challenging in South Africa.

Rehabilitation stakeholders play an important role in accessing and providing rehabilitation within the health care sector. A Cochrane study^18^ discovered that in the context of research, there are different definitions of what rehabilitation means and entails. A uniform understanding of what rehabilitation entails, its purpose, and strategies to achieve this purpose, is important to optimize person-centered rehabilitation provision for people with rehabilitation needs. While it is acknowledged that rehabilitation can have different meanings for different stakeholders in different contexts,^19^ it is important to explore these meanings to provide person-centered and contextually relevant rehabilitation. More importantly, a uniform understanding amongst rehabilitation stakeholders can advance the implementation of policy to practice, the implementation and uptake of evidence-based rehabilitation strategies and interventions and can drive prioritized and appropriate research for rehabilitation, as well as funding for such research and implementation strategies.^19-21^

The involvement of stakeholders in prioritizing rehabilitation in health care systems, uniting the rehabilitation sector, implementing policy into practice, providing evidence-based rehabilitation in clinical practice, and ensuring access to rehabilitation highlights the importance of understanding stakeholder perspectives in different contexts. Examining stakeholder perspectives in the South African context will offer insights into how South African rehabilitation stakeholders perceive and have experienced current rehabilitation practices, as well as the factors influencing the delivery of high-quality rehabilitation services. To our best knowledge, no studies have explored or described the landscape of rehabilitation in the South African context. The aim of the current study was thus to examine the perspectives of South African rehabilitation stakeholders on rehabilitation practices in the public health care sector of South Africa. The objectives were to map out how stakeholders define rehabilitation and explore their understanding of the scope, challenges and progress of rehabilitation service delivery and how various social determinants of health shape rehabilitation health outcomes in South Africa. This mapping is important to ascertain whether there is a mutual understanding of rehabilitation and rehabilitation practices among South African rehabilitation stakeholders. A homogenous understanding of rehabilitation is important to develop a shared vision, develop a strategy for change in terms of policy and practice, and advocate for the prioritization of rehabilitation in health systems in LMICs.^20^

## Methods

### Study Design

A descriptive qualitative study, with an interpretive approach, was conducted to explore the perspectives of rehabilitation healthcare provider stakeholders on rehabilitation services in South Africa. We employed a landscape analysis to get a broader understanding of rehabilitation in the South African context, to identify opportunities, emerging issues and trends of rehabilitation practices and to ascertain potential implementable next steps in practice, policy and research.^22^ We were guided by the social determinants of health framework to interpret and analyze the overall findings of this study.^23^ This approach was followed to describe the “who,” “what,” and “where” involved in rehabilitation practices in the public health care sector in South Africa,^24,25^ and to explore how various social determinants of health shape rehabilitation health outcomes.

### Setting

This study was conducted in South Africa, a culturally and geographically diverse country with 9 provinces. This study focused on the public health care sector because most of the population makes use of the public health care sector.

### Population and Sampling

Key informants/stakeholders with a national, provincial, and/or local standing in rehabilitation and disability in South Africa were invited to participate. The sampling process is illustrated in Supplemental File 1.

#### Inclusion and exclusion criteria

Stakeholders could be involved in clinical work, academia, education, policy processes, or management of rehabilitation services. Rehabilitation stakeholders only practising or solely involved in private health care settings were excluded from this study, since the study focused on the public health care sector in South Africa. This study purposively did not include end users of rehabilitation services, as patients’ perspectives will be explored in a separate future study.

#### Sampling

A 2-step sampling process was applied (Supplemental File 1):

##### Step 1: Purposive sampling

Initially, purposive sampling was used to identify 5 potential participants. These stakeholders were deliberately identified based on their knowledge, interest, and willingness to share their experiences about a particular field of interest.^26^ The purposive sample was determined through discussions with researchers within the field of rehabilitation and disability as well as the supervisory team of the PhD candidate at the time. The participants that were identified included a representative from the disability sector, a clinician who was also involved in rehabilitation research at the time, national stakeholders who were involved in policy processes as well as a known academic who has a special interest in the field of rehabilitation with several peer-reviewed publications on disability and rehabilitation services.

##### Step 2: Snowball sampling

Rehabilitation stakeholders who participated via purposive sampling were asked to nominate stakeholders from different sectors and professions who could potentially contribute rich and diverse information regarding rehabilitation in South Africa. This snowballing approach is useful when a population is dispersed and when the required characteristics for the study are not readily available.^27^ We anticipated that peers’ nomination would result in a diverse range of rehabilitation stakeholders with insight into the South African context of rehabilitation.

#### Sample size

The recruitment process in this study aimed to recruit enough participants that would lead to data saturation.^28^ Data saturation was taken as a point where “*no new information added is expected to enhance or change the findings of the study*,”^29^ with specific reference to reaching the study aim/objectives.

### Data Collection Procedures

Semi-structured individual interviews were conducted between January and November 2020, by the main author, face-to-face, telephonically, or online via Zoom (Zoom Video Communications Inc, Version: 5.12.2 (9281)) or Microsoft Teams (Version 1.6.00.376 (64 bit) according to the preference of the participant. Only 1 interview was conducted face-to-face. The rest of the interviews were conducted online (n = 6) or telephonically (n = 5) from the main author’s home. No other parties were present during the interview process except for the interviewer and the participant. An interview guide was developed using international and national literature.^5,6^ The interview guide (Supplemental File 2) was drafted by the main author, checked by a co-author, and externally audited by a senior researcher in the field. Box 1 presents the questions relevant to this manuscript as the interview guide investigated other factors pertaining to rehabilitation (reported elsewhere)^30^ beyond the scope of this paper. Although the interview guide contained specific questions on the different themes and topics, it provided flexibility for other topics to be covered and allowed for a conversation between the interviewer and interviewee.^27^ The interview guide was piloted with 1 participant before the start of the study by the main author. The pilot interview didn’t reveal any necessary changes before starting the study. The interviewer and the pilot interviewee both agreed that the interview guide, flow, and time needed were suitable and in line with the study’s objectives. No repeat interviews were indicated in this study.

**Box 1.** Interview Guide Topics.

Definition of rehabilitation
Core components of rehabilitation
Factors affecting rehabilitation service delivery in South Africa
Rehabilitation Policy (national vs international)
South African rehabilitation practice in comparison with global rehabilitation practice

A short demographic questionnaire was developed by the main author to capture information about the participant’s current role and workplace, years of experience and areas of expertise/experience. The purpose of this questionnaire was to contextualize the findings of the interviews and to monitor and ensure diversity in sampling.

#### Recruitment process

The main author contacted the initial sample and subsequent nominated stakeholders via email to enquire about their willingness and interest to participate in the study. The contact details of the initial sample and subsequent participants were obtained from their colleagues who nominated them or retrieved from university websites. A second email was sent to the participants who agreed to participate in the study. The second email included an information sheet and consent form, which the prospective participants had to read, complete, and return before the start of the interviews, together with a preferred date, time, and mode for the interview. Two weeks were provided for responses between emails, including follow-up emails. If the contacted participants did not respond within 2 weeks of receiving the 2 follow-up emails, the lack of response was accepted as an indication that the participant was not interested or available to participate in the study, resulting in the termination of the recruitment attempts.

All interviews were conducted in English and lasted between 45 and 75 min. All interviews were recorded using a digital recorder or via Zoom or Microsoft Teams (with or without video recording, depending on the participant’s preference) with additional notetaking. The recordings were all de-identified and transcribed by the main author and professional transcribers.

The main author is a female physiotherapist with clinical experience in the private and public health care sectors in hospitals and outpatient settings. The main author did not have any relations with any of the participants, except for one. At the time of the interviews, the familiar participant was known to the main author professionally. The main author introduced herself as a postgraduate student in physiotherapy to all the participants and was transparent about her interest in rehabilitation and her work experience. Rapport was established at the beginning of each interview, whilst the main author encouraged an environment where the participant could feel free to express their honest opinions and feelings but also feel free to refuse to answer questions whenever the participant felt inclined to. The main author had no prior experience or training in qualitative research methods, except for 2 online short courses in qualitative research, reading literature and books about qualitative research and receiving guidance from their supervisors at the time.

The co-authors were involved in the coding process, data analysis and interpretation processes, drafting and critical revision of this paper. All the authors of this paper are female and native South Africans. The co-authors of this paper are academics and researchers within the rehabilitation community in South Africa and globally. They were not involved in the interview process to maintain the anonymity of the participants who may or may not have been familiar with the co-authors in a personal or professional capacity.

### Data Analysis

A summary of the initial data analysis was sent to the participants for feedback, however, only 1 participant provided feedback which was incorporated into the final data analysis. A combination of deductive and inductive processes was used to analyze the data. For the deductive analysis, the main author developed a conceptual framework (Supplemental File 3) based on a review of South African policy documents^15,16^ as well as WHO guidelines.^5,31^ A priori themes and categories were identified, analyzed, and reported on, based on the conceptual framework, as a “theory” on rehabilitation practices already exists on a global and national level. The authors remained open to the emergence of new themes and categories, however, and used an inductive process to code any novel ideas.^32,33^

### Coding

Three authors each coded 2 interview transcripts independently using the conceptual framework. The authors then met, compared codes and categories, and created a combined codebook via consensus. The main author (RM) then proceeded to code all the transcripts using Computer Assisted Qualitative Data Analysis (CAQDAS) software namely, Atlas.ti, version 9. Open coding was used for the identification of new emerging codes and categories which were then added to the codebook.^29,34,35^ This process of investigator triangulation through multiple analyses,^36^ assisted in a broader understanding of the content of the interviews and ensured a more accurate representation of the final theoretical framework.^27,37^ Using Atlas.ti, the main author (RM) created networks of emerging patterns and correlations between the different codes. The coding process entailed identifying codes through labeling words, phrases and/or sentences.^27^ Codes were grouped according to the frequency of occurrence among the interviews, to ascertain which aspects were most highlighted by the participants. Codes were then categorized and grouped under a particular overarching theme. An iterative process was followed with continuous discussion amongst the research team to ensure consensus on the final set of codes and categories.

### Qualitative Quality Criteria

The study applied the criteria proposed by Frambach et al^38^ to ensure quality assurance throughout this qualitative study. These criteria include *credibility, transferability, dependability*, and *confirmability*.^38^ The study attained *credibility* by means of investigator triangulation whereby the main author coded all the transcripts and 2 co-authors independently coded 2 transcripts each. Prolonged engagement contributed to the quality assurance of this study, as interviews were conducted between January 2020and November 2020. The information on the sampling strategy as well as the description of the context of the findings were techniques used to ensure *transferability*. The purposive sampling technique in conjunction with the snowballing effect facilitated the identification of the most appropriate participants to assist in exploring the phenomenon in question. The flexibility of the research team and openness to the process allowed for *dependability* to transpire. The main author kept an audit trail of aspects around decision-making and different processes which formed part of the data collection and analysis process. Constant reflexivity, manually documented in a diary, was an integral part of the data collection and data analysis process. The main author’s peer debriefing with peers and supervisors about data collection, the findings and the results aided in reflexivity. These techniques are consistent with the fourth criterion defined by Frambach et al^38^ namely, *confirmability*.

### Ethical Considerations and Adherence

The protocol for the study was approved by The Health Research Ethics Committee (HREC). The main author obtained written informed consent from each stakeholder before the commencement of the study and verbal consent was audio recorded at the start of the interview. Unique participant reference numbers were used for recordings, transcriptions, and reporting to ensure the confidentiality of participants.

## Results

Forty-six rehabilitation stakeholders were invited/nominated to participate in the study. However, only 12 rehabilitation stakeholders agreed to participate in the study. Of all the nominees, only 1 nominee was not a health care professional (HCP), but a representative from the disability sector. Unfortunately, this nominee could not participate in the study due to unavailability to conduct an interview. This resulted in only HCPs participating in the study. The reasons for non-participation included a lack of response after follow-up emails were sent to the participants who initially indicated their willingness to participate (n = 9), non-response from 19 stakeholders, conflict of interest (n = 1), as well as 3 stakeholders declining to participate due to retirement, involvement in COVID-19-related activities and time constraints respectively. Following the analysis of 12 interviews, the main author, and another author (LM) convened to determine the richness of data and agreed that the data collected was sufficient to answer the research aims/objectives and that data saturation had been reached, which resulted in 2 nominees not being contacted.

### Participant Demographic Information

Table 1 provides the demographic information of the participants, including the area of expertise within the field of rehabilitation, their profession, years of experience and the province in which they were located during the time of the interviews. Many of the participants had more than 1 area of expertise and coincidentally all the participants were qualified HCPs. Most of the participants were physiotherapists (PTs; n = 7), followed by occupational therapists (OTs; n = 4), and then speech, language and hearing therapists (SLTs; n = 1).

**Table 1.** Demographic Information of Rehabilitation Stakeholders.

Participant	Background	Area of expertise	Years of experience in rehabilitation	Province
P1	PT	Clinical/academic	23	WC
P2	OT	Policy	32	Gauteng
P3	PT	Academic	30	WC
P4	PT	Clinical/Policy/Management	18	KZN
P5	ST	Management/Clinical	28	Mpumalanga
P6	OT	Clinical	29	Gauteng
P7	OT	Academic/Policy	50	KZN
P8	OT	Management/Policy	21	EC
P9	PT	Academic	24	Gauteng
P10	PT	Academic	32	WC
P11	PT	Academic	25	WC
P12	PT	Academic/Clinical	5	WC

PT = physiotherapist; OT = Occupational Therapist; ST = speech therapist; WC = Western Cape; KZN = Kwazulu-Natal; EC = Eastern Cape.

### Interview Results

Five main themes emerged which comprised 2 themes derived from the conceptual framework and 3 novel themes identified through the inductive process (Table 2). Categories were applied to further explore 2 of the themes, as indicated in Table 2. Exemplary/illustrative quotes are provided in this section with each participant’s unique reference number to explore the findings, and more illustrative quotes are provided in Supplemental File 4.

**Table 2.** Themes, Categories, and Sub-Categories Identified from the Interviews.

Theme	Categories	Sub-categories
Definition of Rehabilitation	-	-
Core elements of Rehabilitation	Where (setting)	-
	Who (Providers of)	-
	When (Indications for)	-
Challenges that influence access to rehabilitation in South Africa	Health care system factors	Human resources, geographical location of facilities, health care budget, health care accessibility (ability to reach)
	Health care provider factors	Provider attitudes and competencies
	Awareness of rehabilitation	Health literacy and awareness
	Socio-political factors	
Rehabilitation Policy and Governance	-	-
Progress of Rehabilitation in South Africa	Compared to other Low- and Middle-Income Countries	-
	Compared to high-income countries	-

#### Theme 1: Definition of rehabilitation

Participants described rehabilitation as a health care journey that enables the person to reach the desired level of function. Rehabilitation was also described as a whole person approach, that considers person-specific and environmental factors. Participants emphasized rehabilitation as returning to previous roles in family, work and social life and highlighted the importance of reintegration.

“*. . .it includes all the therapy to address your impairments, all the activities that we do to address activity limitations as well as engaging in the environment to address participation and facilitate reintegration.*” (P10)

Some participants also described what rehabilitation is not.

“*And for me disability versus rehabilitation is very separate. Disability is a philosophical, political understanding of somebody with an impairment . . . Whereas rehabilitation is a medical intervention that may continue. . .*” (P3)

While participants acknowledged that the definition of rehabilitation should be considered in the context of policies, and international and academic definitions, they declared their understanding of rehabilitation, based on their personal experiences as indicated in the quotes below.

“*That is my own take on it*” (P4)

“*I’m not going to give you some academic definition*” (P6)

#### Theme 2: The core elements of rehabilitation

##### 2a When is rehabilitation provided?

Participants recognized the need for access to rehabilitation throughout the life course, from children to the elderly. For the participants, rehabilitation is a health care intervention that should start early in the management of a health condition and can continue to chronic care and community-based rehabilitation (CBR). One stakeholder thought that rehabilitation should have an endpoint and does not continue indefinitely.

“. . .*I mean there are many people who need it. From children below average age milestones to older people who might have functional limitation either with movement, with communication and so on and so forth. . .*” *(P2)*

“*Rehab for me is like okay, you’re still going to work towards some of the CBR goals like towards participation and integration and stuff, but it’s, there is usually a point where we have to stop. And that does not mean that CBR stops – it continuous*” *(P4*)

##### 2b: By whom is rehabilitation provided

Participants agreed that the rehabilitation team should comprise a multidisciplinary team of HCPs which includes not only rehabilitation professionals, but also medical doctors, social workers, psychologists, and community members. As mentioned by the participants, these community members form a core part of the rehabilitation team and could comprise community-based workers, peers, volunteers, family, and caregivers.

“*It is the physio, the OT, the speech, the audio, the MOP* (medical orthotist and prosthetist), *the psychologists, the social workers. Sometimes even we have got the rehabilitation doctor. . .*” *(P8)*

“*And we don’t see it just limited to OT, physio, speech, language, and audiology. We see it, even the part that the family plays, contribute to rehabilitation, and in all community. . ..*” *(P2)*

##### 2c: Where is rehabilitation provided?

Participants emphasized that, according to policy, rehabilitation can and should be provided throughout all the levels of health care from acute stages (hospital-based in-patient rehabilitation) and post-discharge (out-patient rehabilitation). Rehabilitation was thought not to be confined to health care centers, but needs to be integrated into schools, old-age homes and community centers. However, participants recognized that the provision of rehabilitation at specialized facilities is dependent on the availability of resources in different settings

“*. . . most rehabilitation happens in hospital and primary health care facilities. I know that we do it at varying degrees depending on resources and such like. There is quite a lot of rehab that happens in an outreach context, so some patients we do home visits, some do visits to institutions, centres and old age homes and schools. . .*” *(P5)*

#### Theme 3: Challenges affecting rehabilitation services in South Africa

The participants highlighted that the South African rehabilitation sector faces several challenges on various levels. A particular challenge which was emphasized was access to health care, which was thought to be influenced by several contextual factors comprising health care system factors, health care provider factors, patient-specific factors, and socio-political factors.

##### 3a: Health care system organizational factors

In terms of the health care system factors, the participants highlighted the significant limitations pertaining to the capacity and distribution of human resources especially in rural areas and at PHC level.

“*The capacity at different levels of care. There is not sufficient numbers of rehabilitation workers, just service at the different levels of care. Also, the complement of rehabilitation professionals is also not always appropriate at the different levels of care . . . we don’t always see what the need of the community at that level is. Is what I have got appropriate?*” *(P3)*

“*. . .the rural areas are very understaffed. . . the highest level of disability is where the lowest level of therapists are, it is just frustrating*” *(P5)*

Participants reported that access to rehabilitation is also limited because of the shortage of health care facilities, again especially in rural/remote areas and at PHC level which necessitates clients to travel to district and tertiary facilities that are far away from them resulting in increased out-of-pocket expenditure to an often already financially restrained household. Furthermore, a lack of transport for providers to perform outreach activities where rehabilitation services are unavailable was reported to challenge access to rehabilitation as well.

“*Transport is another big one. In the rural contexts where I work a lot, many times a person with a mobility disability particularly, isn’t actually able to make use of so-called public transport . . . but often the cost of hiring a vehicle, private vehicle is almost the entire value of the monthly pension*” *(P6)*

“*We have started those outreaches; they are not consistent because even the therapists themselves have challenges when it comes to transport to go to those clinics*” *(P8)*

Included in health care system challenges, are financial and/or budgetary constraints that impact the availability of proper infrastructure, and provision of assistive devices (ADs) as reported by our participants. Despite the awareness of policy on ADs, participants noted that access to ADs is further limited due to backlogs, the lack of appropriate ADs or the unavailability of ADs.

“*But there isn’t enough budget given to provinces for assistive devices. And also we can . . . but we don’t, we tend to look for the cheapest rather than what is ideal for the patient just so we can give as many patients assistive devices as possible and rather give them something than nothing.*” (P5)

##### 3b: Provider attitudes, competence, and skills

Participants raised a concern about the attitudes and competence of rehabilitation professionals that could impact the quality of rehabilitation provided as well as the ability to address the unique needs of the community that is particularly evident in public health care, which potentially stems from inadequate undergraduate training.

“*. . . rehab professionals are not trained to deal with the crises you find in rural areas. The students at universities aren’t trained properly.*” (P7)

##### 3c: Awareness of rehabilitation services and health literacy

The participants also mentioned that a lack of awareness on the patient’s side as well as ineffective referral systems from the providers’ side, coupled with a lack of knowledge of the value of rehabilitation services, can lead to rehabilitation services not being accessed.

“*This link to access is also health literacy that*’*s poor because if you don*’*t know, you don*’*t. If you*’*re not referred, most people won*’*t ask because they don*’*t know*” *(P11)*

##### 3d Socio-political context

Additional socio-political and environmental factors such as the physical environment, weather conditions, crime, and political riots as well as poverty and unemployment, were also highlighted by the participants which create barriers to accessing rehabilitation services. Participants shared that in the context of patients’ financial limitations, the provision of basic needs takes preference over accessing rehabilitation.

“*People are dealing with issues where they have to prioritise what they use their money for so they will rather eat or give food and sustenance to their families rather than to go for health care.*” (P1)

“*A huge barrier for people in wheelchairs, the wheelchair users, was crime. . . I don’t know what the flipside is, because as dangerous as it is for patients to walk to the clinics, it is for the professionals to go to their homes.*” (P3)

“*And then there*’*s weather plays a big role. So um, in our area, if it was a rainy day, the roads are very muddy. They, the, the taxis wouldn*’*t run on rainy days and sometimes the rivers would overflow so they won*’*t be able to cross the rivers.*” *(P12)*

#### Theme 4: Governance and policy

Participants acknowledged that South Africa has adequate policies for rehabilitation. However, the implementation of policies remains a challenge. Participants voiced their frustration regarding the lack of engagement and participation from policymakers to prioritize rehabilitation services and to ensure the implementation of policies into practice.

“*. . .but the problem is not policy. The problem is implementation. And resources.*” (P11)

#### Theme 5: Progress of rehabilitation in South Africa

Participants were optimistic about the progress that South Africa has made in developing access to rehabilitation, despite the limited resources and many challenges experienced. When compared to other LMICs, participants mentioned South Africa’s progress relating to policy, funding, awareness, human resources, and equipment, although there is ample room for development when compared to high-income countries (HICs).

“*I think the number of physios we have in South Africa; the total is more than a combined total for, for most of these countries. . . When I say low-income countries, we*’*re not just talking about Africa because we also have students from India, Pakistan, we have. . . and then they also have shortage. . . in terms of human resource*” *(P9)*

“*But as far as the WHO action plan is concerned, we are one of the countries with an established rehabilitation service. You have a national presence, you have a provincial presence, and rehabilitation in South Africa is a state function. It is funded by the state. . .*” (P2)

“*. . . if we have to go to the rehab unit in. . . the one in Cape Town or the one in Tshwane I’d say, we compare very well* [ to the UK] *and. . . OK, when I say very well, I don*’*t mean it*’*s exactly the same, but yes, I think our patients are getting, (uh) good rehab.*” (P9)

## Discussion

This study aimed to investigate the landscape of rehabilitation in South Africa by gathering the perspectives of local rehabilitation stakeholders on the definition of rehabilitation and current practices in the public health care sector. The main findings of the study illustrated that the participants shared a common philosophy on rehabilitation and identified several multi-domain context factors that challenge the effective provision of rehabilitation in the South African context. These factors clearly show how the interaction between social determinants of health, such as environmental, social, and economic factors, as well as policy implementation, can affect the health outcomes of individuals requiring rehabilitation in South Africa. Despite these challenges, the participants were encouraged by the progress made in South Africa to develop good quality rehabilitation policies, although the focus should now shift to implementing these policies.

### Definition and Core Elements of Rehabilitation

Participants each shared their unique interpretation of what rehabilitation entails. For participants, rehabilitation encompasses a holistic health care approach that endeavors to enable a person to reach a status of optimized function and participation within that person’s unique personal, social, and environmental factors. Rehabilitation, provided over the life course, was seen as a health care journey in which the person in need of rehabilitation is accompanied and supported by a multidisciplinary team of health care providers and community members at all levels of care. For rehabilitation to be holistic, it needs to cross the border of health care facilities to be available where people live, enjoy leisure and work.^39^ Our participants’ description of the core elements of rehabilitation is congruent with WHO guidelines^40^ as well as South African health care policies,^15,16^ however, it focusses uniquely on the influence of the socio-political and environmental factors faced on an individual’s journey within the South African context.

The congruence in the stakeholder definitions and descriptions of rehabilitation is encouraging, since Wade^19^ argues that the focus should not merely be on defining rehabilitation, but be united on a common understanding within the context. Neill et al^20^ emphasize the importance of a shared understanding of rehabilitation but highlight the significance thereof with regards to government/ policymakers and the rehabilitation sector which could lead to prioritization of rehabilitation services. Our participants shared a similar rehabilitation philosophy, and it is important that this philosophy is shared by rehabilitation policymakers.

### The Role of Policy in the Provision of Rehabilitation

Translation of policy into practice was an important consideration for participants to optimize rehabilitation practices in the South African context. The participants agreed that making concerted efforts to prioritize rehabilitation and implement policies will have a positive impact in providing resources for rehabilitation and enhancing access to rehabilitation. Indeed the South African National Rehabilitation Policy (NRP)^15^ as well as the Framework and Strategy for Disability and Rehabilitation services (FSDR),^16^ provide direction on the implementation of rehabilitation policy. Both the NRP and the FSDR have been reviewed in the literature. Mji et al^41^ illustrated more than a decade ago that the objectives of the NRP had not been met at the time. Similarly, a recent review by Hussein El Kout et al^42^ revealed many barriers to the implementation of the FSDR mission, including actor dynamics, insufficient resources, a rushed process, poor record-keeping, inappropriate leadership, negative attitudes of staff members and insufficient monitoring.^42^ The objectives of both these legislative documents speak to improving accessibility, intersectoral collaboration, human resource development, monitoring and evaluation strategies of rehabilitation and disability services.^15,16^ Although the objectives of these documents are congruent with what the participants in our study highlight as important considerations for optimizing rehabilitation in South Africa, it is evident that these goals have not been met, even though it’s been highlighted almost 10 years ago. Consequently, there is a need to investigate the implementation delays for the well-aimed objectives of the NRP as well as the FSDR, to narrow the gap between policy and practice.

It has been suggested that the inability to translate policy into practice for rehabilitation may be the lack of understanding of the value of rehabilitation services.^43^ Possible solutions to this lack of understanding are to advocate for and provide empirical evidence for the importance and value of rehabilitation. Calls have also been made to improve research and data collection methods to provide better key indicators as well as priority health outcomes for rehabilitation and to get a better picture of the utilization of rehabilitation services by PWDs.^43,44^ Therefore there is a need to conduct rehabilitation program evaluations^41^ in order to provide appropriate and reliable rehabilitation data^43^ and to assist in informed decision-making by policymakers. Implementation of policy will play an important role in addressing the significant need for rehabilitation and by unlocking resources to address the challenges associated with rehabilitation practices.

### Factors Affecting Access to Rehabilitation Services

Several multidomain contextual factors that challenge access to rehabilitation services in South Africa have been identified by the participants. These domains comprise health care system factors, patient-specific factors, health care provider factors and socio-environmental factors. These factors correspond with contextual factors involved with implementing complex interventions.^45^ In addition, these factors also correlate with the 5 dimensions of access namely, approachability, affordability, acceptability, availability and accommodation as well as appropriateness.^46^ Evidently from the findings of our research, the limitations in access to rehabilitation services cannot be fully understood without providing the context. Lau et al ^45^ emphasize the importance of understanding context when implementing complex interventions, as barriers and facilitators change, and they interact with each other. For example, people in rural and resource-limited areas often incur additional transport costs when they have an impairment that requires the use of an AD. They would also require a caregiver to accompany them in traveling long distances over poorly kept roads to a health care facility where there may not be any rehabilitation professionals. The above example illustrates that the needs of PWDs can be complex^47^ and that rehabilitation provision needs to be person- and context-specific. It is evident from our results that social determinants of health play a significant role in rehabilitation service delivery in South Africa. MacLachlan et al^48^ state that access to health care “can neither be universal nor equitable if it is less accessible to some sections of society than to others.” The findings of the current study emphasize that action must be taken to develop multisectoral collaboration to address the multidomain context factors to optimize access to rehabilitation for people with rehabilitation needs. Some of these actions may include interdisciplinary rehabilitation approaches, collaboration with PWDs, communities, families and community leaders to promote disability rights, collaboration with government toward the recognition of rehabilitation as a human rights issue as well as providing support in terms of designated transportation for PWDs.^49^

### The Impact of Human Resource Development on Access to Rehabilitation

Our participants highlighted that limitations in human resources are a major challenge in terms of access to rehabilitation. Both the NRP and the FSDR mention human resource development as one of the goals of the respective policies. Evidence from the literature suggests that very little human resource development has been done as the workforce capacity has been found to be low and inequitable in 3 rural provinces in South Africa.^50^ Burger and Christian^11^ reported that access to health care services in terms of availability, acceptability and affordability remains very low and inequitable in South Africa—despite health care reforms—as only 53% of their study respondents had full access to health care. The authors found that availability and affordability of health care services are highly challenged by the remoteness of certain areas. This amplifies the findings by Conradie et al^50^ who reported that the lowest rehabilitation workforce was found in rural areas and at PHC which ultimately means that those who need rehabilitation the most have the poorest access to rehabilitation. Although the South African National DoH has endeavored to improve access to health care through the PHC reengineering initiative,^51-53^ it is evident from reports in the literature as well as the findings in our study, that rehabilitation is severely challenged at PHC level, which means that access to rehabilitation services for the most vulnerable populations is limited.

In the South African context, with the roll-out of the NHI, the time is opportune to engage in collaborative efforts with the different sectors to strategically prioritize an implementation plan to ensure access to rehabilitation. Prioritizing rehabilitation (in policy and practice) will enable the personal, community, economic and societal benefits rehabilitation can provide. Such prioritization is important since, for people with rehabilitation needs, a lack of access to rehabilitation can result in worse health outcomes, and deterioration in function and could add to the disability-poverty nexus when reintegration and participation in education and employment are hampered.^44,54,55^ Despite the many challenges that the rehabilitation sector faces in the South African context, participants generally felt that South Africa’s rehabilitation landscape has made good progress when compared to other LMICs and in some cases potentially on par with high-income countries. They were hopeful that this progress would continue.

### Strengths and Limitations

This study adds to the body of knowledge on the understanding of rehabilitation practices and challenges for the provision of and the progress of rehabilitation in the South African context. A strength of our study is that the findings are based on a multidisciplinary perspective and reiterate similar challenges across professions and levels of health care. The findings confirm a commonality in defining rehabilitation and a vision for rehabilitation amongst key rehabilitation stakeholders, which is important in driving the priorities for rehabilitation. Our findings echo many of the known challenges for health care provision in LMICs and therefore reiterate the need for implementation of policy to practice providing equitable services to those who need it. To achieve this common goal of providing quality and equitable rehabilitation, a collaborative and multisectoral approach is required.

Several study limitations need to be considered when interpreting the study findings. The participants were all HCPs, of which the majority were physiotherapists, and mostly from the Western Cape province. Notably, only 5 provinces out of the total of 9 had representation, due to the sampling method applied. A more heterogeneous group of participants could provide further insights into other province-specific nuances and profession-specific perspectives that were not identified in this study which may have had an impact on achieving data saturation and profession-specific analysis. We therefore recommend that future studies of a similar nature should use stratified sampling to ensure that all provinces are represented. We also acknowledge that the background of the different participants may influence their interpretation and perceptions of rehabilitation practices in the South African context. It is therefore recommended that a broader demographic of the participants is established by inquiring about the participants’ country of origin, their native language, race, gender and age. Additionally, a more diverse body of rehabilitation stakeholders can be included, for example, apart from academics, policymakers, and clinicians, to also include rehabilitation representatives, NGO representatives and patients. Patients’ perspectives will however be explored in a follow-up qualitative study to align with the global shift toward value-based and patient-centered care.

Challenges were experienced in the recruitment of participants, since the study commenced shortly before the COVID-19 pandemic in South Africa and continued throughout the early stages of the pandemic. Many of the invited participants did not respond or could not participate due to COVID-19 related commitments or due to prioritizing personal or work commitments in a time when so much was unknown about the virus and its impact and implications.

## Conclusion

Rehabilitation stakeholders in this study shared a similar philosophy on how rehabilitation is defined and what rehabilitation practices in the South African context entail. The findings indicate several contextual factors including various social determinants of health, that need to be addressed to ensure equitable access to quality rehabilitation services in the South African context considering the implementation of the NHI. There is an impetus for policy makers to provide resources for the implementation of existing, adequate policies to address the contextual factors that have been reported for decades. Participants were encouraged by the progress made in the rehabilitation agenda, albeit slow. Further research should explore the rehabilitation needs of end-users, together with collaborative research for priority setting on the translation of policy to practice ensuring equitable and quality rehabilitation service delivery.

## Supplemental Material

sj-docx-1-inq-10.1177_00469580241271973 – Supplemental material for The Rehabilitation Landscape in a Low-to-Middle-Income Country: Stakeholder Perspectives and Policy Implications—A Qualitative Study Supplemental material, sj-docx-1-inq-10.1177_00469580241271973 for The Rehabilitation Landscape in a Low-to-Middle-Income Country: Stakeholder Perspectives and Policy Implications—A Qualitative Study by Rentia Amelia Maart, Gubela Mji, Linzette Deidré Morris and Dawn Verna Ernstzen in INQUIRY: The Journal of Health Care Organization, Provision, and Financing

sj-docx-2-inq-10.1177_00469580241271973 – Supplemental material for The Rehabilitation Landscape in a Low-to-Middle-Income Country: Stakeholder Perspectives and Policy Implications—A Qualitative Study Supplemental material, sj-docx-2-inq-10.1177_00469580241271973 for The Rehabilitation Landscape in a Low-to-Middle-Income Country: Stakeholder Perspectives and Policy Implications—A Qualitative Study by Rentia Amelia Maart, Gubela Mji, Linzette Deidré Morris and Dawn Verna Ernstzen in INQUIRY: The Journal of Health Care Organization, Provision, and Financing

sj-docx-3-inq-10.1177_00469580241271973 – Supplemental material for The Rehabilitation Landscape in a Low-to-Middle-Income Country: Stakeholder Perspectives and Policy Implications—A Qualitative Study Supplemental material, sj-docx-3-inq-10.1177_00469580241271973 for The Rehabilitation Landscape in a Low-to-Middle-Income Country: Stakeholder Perspectives and Policy Implications—A Qualitative Study by Rentia Amelia Maart, Gubela Mji, Linzette Deidré Morris and Dawn Verna Ernstzen in INQUIRY: The Journal of Health Care Organization, Provision, and Financing

sj-docx-4-inq-10.1177_00469580241271973 – Supplemental material for The Rehabilitation Landscape in a Low-to-Middle-Income Country: Stakeholder Perspectives and Policy Implications—A Qualitative Study Supplemental material, sj-docx-4-inq-10.1177_00469580241271973 for The Rehabilitation Landscape in a Low-to-Middle-Income Country: Stakeholder Perspectives and Policy Implications—A Qualitative Study by Rentia Amelia Maart, Gubela Mji, Linzette Deidré Morris and Dawn Verna Ernstzen in INQUIRY: The Journal of Health Care Organization, Provision, and Financing
