# Supplementary material for: The Rehabilitation Landscape in a Low-to-Middle-Income Country: Stakeholder Perspectives and Policy Implications—A Qualitative Study
Source: Inquiry. 2024 Oct 7;61:00469580241271973. doi: 10.1177/00469580241271973 (PMC11459787; doi:10.1177/00469580241271973)
Supplement: sj-docx-1-inq-10.1177_00469580241271973 – Supplemental material for The Rehabilitation Landscape in a Low-to-Middle-Income Country: Stakeholder Perspectives and Policy Implications—A Qualitative Study [file sj-docx-1-inq-10.1177_00469580241271973.docx]

# Appendix 1

Organogram illustrating the sampling process that was followed.
