# Supplementary material for: The Rehabilitation Landscape in a Low-to-Middle-Income Country: Stakeholder Perspectives and Policy Implications—A Qualitative Study
Source: Inquiry. 2024 Oct 7;61:00469580241271973. doi: 10.1177/00469580241271973 (PMC11459787; doi:10.1177/00469580241271973)
Supplement: sj-docx-2-inq-10.1177_00469580241271973 – Supplemental material for The Rehabilitation Landscape in a Low-to-Middle-Income Country: Stakeholder Perspectives and Policy Implications—A Qualitative Study [file sj-docx-2-inq-10.1177_00469580241271973.docx]

# Supplementary File 2: Interview GUIDE

1. **The researcher introduces herself, greets the participant, ensures that they are still willing to participate and confirms their consent on the recording.**
2. **Opening discussion**
   1. How did you become involved in rehabilitation, where did it all start?
   2. What does your current role entail?
   3. What other team members do you work alongside with?
   4. Have you worked with anyone internationally pertaining to physical rehabilitation and can you tell me more about that collaboration?
   5. You have a clear interest in and/or passion for rehabilitation. In your opinion, how would you define rehabilitation?
3. **Main discussion part I**
   1. Based on your experience and background in rehabilitation, could you tell me more about rehabilitation in South Africa in terms of where it is delivered, by whom, why and when?
   2. And in relation to PHC?
   3. what are the challenges South Africans are currently facing in terms of **accessing** and **providing** rehabilitation?
   4. Are you familiar with national policies related to rehabilitation and which ones?
   5. If yes to #4, what are your views on our national policies (e.g., NDP, the national rehabilitation policy, Disability policy guideline)?
   6. Have you any insight into the process of the allocation and management of budgets related to rehabilitation? If so, please elaborate.
   7. Various initiatives and guidelines have been proposed by eg WHO such as the global disability action plan 2014-2021, Rehab 2030: a call for action; Rehabilitation in Health Systems: a guide for action (2019). How would you say, South Africa as a country is doing in relation to these initiatives?
   8. How do we compare to other LMIC as well as high-income countries?
   9. How do you think the NHI will affect the delivery of rehabilitation in the future?
4. **Main discussion part II**
   1. How would you define the concept of “value” in general?
   2. More and more research is being done on the “value” of healthcare and the underlying premise is that healthcare should move away from volume-based towards value-based services. What factors do you think are important to consider when the concept of “value” in terms of Rehabilitation, is explored?
5. **Wrap up**
   1. What can **healthcare professionals** do to improve service delivery in order to align with the various initiatives from the WHO? E.g. global disability action plan 2014-2021; Rehab 2030 a call for action, “Health for all”
   2. What can **government/ policy makers** do to improve our service delivery?
   3. Can you identify other stakeholders that may be able to contribute to this study in a way that we can explore the structure of rehabilitation even further?
6. **Closing**
   1. Thank you for your time
