# Supplementary material for: The Rehabilitation Landscape in a Low-to-Middle-Income Country: Stakeholder Perspectives and Policy Implications—A Qualitative Study
Source: Inquiry. 2024 Oct 7;61:00469580241271973. doi: 10.1177/00469580241271973 (PMC11459787; doi:10.1177/00469580241271973)
Supplement: sj-docx-4-inq-10.1177_00469580241271973 – Supplemental material for The Rehabilitation Landscape in a Low-to-Middle-Income Country: Stakeholder Perspectives and Policy Implications—A Qualitative Study [file sj-docx-4-inq-10.1177_00469580241271973.docx]

**SUPPLEMENTARY FILE 4 –** Additional illustrative quotes aligned with the main themes and categories that emerged from the interviews with rehabilitation stakeholders.

### Theme 1: Definition of Rehabilitation

*“… journeying with that person to be able to get their life back – whatever that means – and it’s very much linked to function, their day-to-day what floats their boat, what they do. What is meaningful for them”* (P6)

*“By definition I think… that we are restoring maximum independent functioning in all areas of participation in social and education and working life, after where that participation is hampered in some kind of way by any kind of disability.”* (P5)

*“…it includes all the therapy to address your impairments, all the activities that we do to address activity limitations as well as engaging in the environment to address participation and facilitate reintegration.”* (P10)

Two participants also described what rehabilitation is not.

*“And for me disability versus rehabilitation is very separate. Disability is a philosophical, political understanding of somebody with an impairment … Whereas rehabilitation is a medical intervention that may continue...”* (P3)

*“I would put rehabilitation as the… more the phase of the whole process but I definitely wouldn’t put CBR into rehab, that’s more development*.” (P4)

Participants declared their understanding of rehabilitation, based on their personal experiences as indicated in the quotes below.

*“… without looking at WHO, CPT … all of those definitions…”* (P3)

*“That is my own take on it”* (P4)

*“I’m not going to give you some academic definition”* (P6)

*“… pretty broad but that is how I see it.”* (P7)

### THEME 2: The core elements of rehabilitation

#### 2a When is rehabilitation provided

*“…I mean there are many people who need it. From children below average age milestones to older people who might have functional limitation either with movement, with communication and so on and so forth...”* (P2)

*“… I think it mostly happens in hospitals in an acute stage, it happens in the therapist’s room in the non-acute at a more chronic state and for people who have a disability, it happens in the homes”* (P5)

*“Rehab for me is like okay, you’re still going to work towards some of the CBR goals like towards participation and integration and stuff, but it’s, there is usually a point where we have to stop. And that does not mean that CBR stops – it continuous”* (P4)

#### 2b: By whom is rehabilitation provided

*“It is the physio, the OT, the speech, the audio, the MOP* (medical orthotist and prosthetist)*, the psychologists, the social workers. Sometimes even we have got the rehabilitation doctor…*(P8)

*“And we don’t see it just limited to OT, physio, speech, language, and audiology. We see it, even the part that the family plays, contribute to rehabilitation, and in all community….”* (P2)

*“…there are people like the mid-level workers and the peer supporters that are excellent at CBR...”* (P4)

#### 2c: Where is rehabilitation provided

*“We are located as NHI - as the policy states-, and as WHO regulations state, that we are at all levels of care. So, we are at tertiary level of care, we are at secondary level of care, we’re at intermediary level of care, and then we are community-based level or your entry-based level of care, and community-based levels of care.”* (P3)

*“… most rehabilitation happens in hospital and primary healthcare facilities. I know that we do it at varying degrees depending on resources and such like.* *There is quite a lot of rehab that happens in an outreach context, so some patients we do home visits, some do visits to institutions, centres and old age homes and schools...”* (P5)

*“We don't have enough rehabilitation units in the country, the whole country 'cause we have one big one in Cape and one big one in Tshwane, Gauteng and the rest are just small beds here and there...”* (P9)

### THEME 3: Challenges affecting rehabilitation services in South Africa

#### 3a: Healthcare system organisational factors

*“The capacity at different levels of care. There is not sufficient numbers of rehabilitation workers, just service at the different levels of care. Also, the complement of rehabilitation professionals is also not always appropriate at the different levels of care … we don’t always see what the need of the community at that level is. Is what I have got appropriate?”* (P3)

*“…the rural areas are very understaffed… the highest level of disability is where the lowest level of therapists are, it is just frustrating”* (P5)

*“We still have communities that do not have accessible rehab services near them and that have to go either to a district- or you know, regional hospital to access services – access remains a challenge.”* (P2)

*“And even now we still don’t have a primary healthcare level... As a result, now we have this therapist, this big number of therapists at tertiary hospitals, and then the patients only go there when they are critical and they need to be stabilised, and once they are stabilised, they have to be down referred to the district hospital. And now when they get to the district hospital, they find no therapists there.”* (P8)

*“Transport is another big one. In the rural contexts where I work a lot, many times a person with a mobility disability particularly, isn’t actually able to make use of so-called public transport … but often the cost of hiring a vehicle, private vehicle is almost the entire value of the monthly pension”* (P6)

*“We have started those outreaches; they are not consistent because even the therapists themselves have challenges when it comes to transport to go to those clinics”* (P8)

*“...and then, in terms of equipment, there was just like a big overall budget for hospital for equipment. But because it was an overall one, things for the doctors and nurses were always prioritized. And then in terms of the process, that was the other problem so. There was a lot of red tape and limitations and, and I think the main thing is because not a lot of rehab equipment is on tender.”* (P12)

*“But there isn’t enough budget given to provinces for assistive devices. And also we can ... but we don’t, we tend to look for the cheapest rather than what is ideal for the patient just so we can give as many patients assistive devices as possible and rather give them something than nothing.”* (P5)

*“I was part of the XXX panel for the revision of the standardisation and provision of assistive devices and I’m very upset about the outcome of that... it should have been revised ages ago etc. etc. There is nothing wrong with the policy as per sè, but what is lacking is the monitoring and evaluation component and then obviously a budget component”* (P4)

#### 3b: Provider attitudes, competence, and skills

*“Lest you have a high turnover of therapists going through the system, they don’t understand the nuances, they don’t understand the cultural barriers... the dynamic barriers, geographical barriers .... so the focus of rehab is often ineffectual, it’s still very medical-model”* (P4)

*“Health professionals, especially rehab professionals, they are not accountable… so that's the first thing that we need to become. We need to, you know, enhance our professionalism, and think of ourselves as being important.”* (P11)

*“… rehab professionals are not trained to deal with the crises you find in rural areas. The students at universities aren’t trained properly.”* (P7)

#### 3c: Awareness of rehabilitation services and health literacy

*“This link to access is also health literacy that's poor because if you don't know, you don't. If you're not referred, most people won't ask because they don't know”* (P11)

*“…because of the lack of knowledge of rehab, I think especially amongst the Primary Healthcare workers like nurses etc so they would only be referred once it's been a problem for really, really, long and they then decide that they need to go to the hospital because it's become that bad.”* (P12)

#### 3d Socio-political context

*“People are dealing with issues where they have to prioritise what they use their money for so they will rather eat or give food and sustenance to their families rather than to go for healthcare.”* (P1)

*“Rehab should also be advocating around provision of household amenities; it should be advocating around the types of housing designs etc. that are available at community level. I mean the average RDP house in South Africa is not exactly wheelchair friendly. The average outdoor toilet whether it is a flushing toilet, or a traditional toilet is not remotely accessible. So if we want to get real about rehab I think we need to move way out of the hospital.”* (P6)

*“A huge barrier for people in wheelchairs, the wheelchair users, was crime… I don’t know what the flipside is, because as dangerous as it is for patients to walk to the clinics, it is for the professionals to go to their homes.”* (P3)

*“… and every now and then there would be riots and things making it unsafe to go…* (P5)

*“And then there's weather plays a big role. So um, in our area, if it was a rainy day, the roads are very muddy. They, the, the taxis wouldn't run on rainy days and sometimes the rivers would overflow so they won't be able to cross the rivers.”* (P12)

*“The infrastructure in terms of – they might be able to walk – but they have to walk long distances over very poor geographical surfaces – and that means that they can’t always get to where they need to get to.”* (P1)

### THEME 4: Governance and policy

*“Because the policy is there, but there really isn’t anything ... to put into practice as has been intended.”* (P5)

*“The best thing that they could do would be to get out and let other people get in. Because the present government policy makers are ... very poor.”* (P7)

*“…but the problem is not policy. The problem is implementation. And resources.”* (P11)

*“I wish those people who are managing… can have some physical impairments and understand one because most of the time you have to go through rehab or having a very close family member go through rehab for them to actually see. So, it doesn't look like people who are sitting with the basket of money understand the importance of quality of life for our population group that has…”* (P9)

### THEME 5: Progress of Rehabilitation in South Africa

*“I think the number of physios we have in South Africa; the total is more than a combined total for, for most of these countries… When I say low-income countries, we're not just talking about Africa because we also have students from India, Pakistan, we have… and then they also have shortage… in terms of human resource”* (P9)

*“But as far as the WHO action plan is concerned, we are one of the countries with an established rehabilitation service. You have a national presence, you have a provincial presence, and rehabilitation in South Africa is a state function. It is funded by the state…”* (P2)

*“In Africa, South Africa is considered as a benchmark and at national we host other sister countries like Namibia. They have come to South Africa for benchmarking, Zimbabwe they have come to South Africa for benchmarking, Botswana that has come to South Africa for benchmarking. Others will just ask for our documents like guidelines and policies for benchmarking.”* (P2)

“*When it comes to knowledge, it comes to equipment I would say they* [UK] *compare very well to most of our rehab facilities that are dedicated like your final rehab, our rehab units… but they do not compare very well to our hospital-based rehab services and our community-based rehab services. Because of the challenges we spoke about, the transport and even at a Community Centre, the equipment we have at the clinic, but if we have to go to the rehab unit in… the one in Cape Town or the one in Tshwane I’d say, we compare very well and... OK, when I say very well, I don't mean it's exactly the same, but yes, I think our patients are getting, Uh… good rehab.”* (P9)
